# The Impact of COVID-19 on People Living with HIV-1 and HIV-1-Associated Neurological Complications

**DOI:** 10.3390/v15051117

**Published:** 2023-05-05

**Authors:** Debashis Dutta, Jianuo Liu, Huangui Xiong

**Affiliations:** Department of Pharmacology and Experimental Neuroscience, College of Medicine, University of Nebraska Medical Center, Omaha, NE 68198-5880, USA

**Keywords:** SARS-CoV-2, COVID-19, HIV-1, PLWH, HAND, neuroinflammation, inflammasomes, microglia

## Abstract

The severe acute respiratory syndrome coronavirus-2 (SARS-CoV-2) is the causative pathogen of the coronavirus disease 2019 (COVID-19) pandemic, a fatal respiratory illness. The associated risk factors for COVID-19 are old age and medical comorbidities. In the current combined antiretroviral therapy (cART) era, a significant portion of people living with HIV-1 (PLWH) with controlled viremia is older and with comorbidities, making these people vulnerable to SARS-CoV-2 infection and COVID-19-associated severe outcomes. Additionally, SARS-CoV-2 is neurotropic and causes neurological complications, resulting in a health burden and an adverse impact on PLWH and exacerbating HIV-1-associated neurocognitive disorder (HAND). The impact of SARS-CoV-2 infection and COVID-19 severity on neuroinflammation, the development of HAND and preexisting HAND is poorly explored. In the present review, we compiled the current knowledge of differences and similarities between SARS-CoV-2 and HIV-1, the conditions of the SARS-CoV-2/COVID-19 and HIV-1/AIDS syndemic and their impact on the central nervous system (CNS). Risk factors of COVID-19 on PLWH and neurological manifestations, inflammatory mechanisms leading to the neurological syndrome, the development of HAND, and its influence on preexisting HAND are also discussed. Finally, we have reviewed the challenges of the present syndemic on the world population, with a particular emphasis on PLWH.

## 1. Introduction

The world is in the middle of a debilitating coronavirus disease 2019 (COVID-19) pandemic, which overlaps with the acquired immunodeficiency syndrome (AIDS) epidemic [1,2,3]. The causative agent for COVID-19 is severe acute respiratory syndrome coronavirus-2 (SARS-CoV-2), and for AIDS, it is human immunodeficiency virus type-1 (HIV-1) [4,5,6,7,8]. Worldwide, SARS-CoV-2 infections exceed 758 million, with more than 6.8 million deaths due to COVID-19 [9]. However, there is a total of 84.2 million people infected with HIV-1 and 40.1 million deaths from AIDS-related illnesses. In 2021, the total number of people living with HIV-1 (PLWH) was 38.4 million, with 1.5 million new infections and more than 650 thousand deaths [10,11]. The coinfection of SARS-CoV-2 and HIV-1 results in SARS-CoV-2/COVID-19/and HIV-1/AIDS co-pandemic or syndemic [12,13]. It was presumed that PLWH are at higher risk of SARS-CoV-2 infection and resultant outcomes owing to their dysregulated immunity and inflammatory conditions, but there is a lack of clear consensus [13,14,15,16,17,18]. A paucity of higher risk may be possibly due to combined antiretroviral treatment (cART) regiments with suppressed viral loads and nearly immune reconstituted status. However, worldwide reports revealed severe clinical presentations, and the increased morbidity and mortality of SARS-CoV-2 infection in PLWH compared to people without HIV-1 [19,20,21,22,23]. In addition to respiratory syndromes, COVID-19 induces neurological manifestations such as headache, confusion, impaired consciousness, anosmia, ageusia, meningoencephalitis, neuropsychiatric disorder and others [24,25,26,27]. Thus, the emergence of COVID-19-associated neurological manifestations may have cumulative neurological manifestations in PLWH and people who have progressed to HIV-1-associated neurocognitive disorders (HAND) [28,29].

HIV-1 invasion into the central nervous system (CNS) was recognized early in the HIV-1 epidemic, and brain infections have been well-studied and characterized, as are viral proteins and their neurotoxicity [30,31,32]. Emerging evidence indicates that SARS-CoV-2 invades the CNS and modulates the host immune responses, causing neurological manifestations [33,34,35]. It is noticed that post-acute sequelae of SARS-CoV-2 (PASC) or Long-COVID has an adverse impact on PLWH, especially in people with HAND [36,37]. This review portrays recent advances in neurological manifestations imposed by SARS-CoV-2/COVID-19 on PLWH with or without HAND. It covers the similarities and differences between SARS-CoV-2 and HIV-1; delineates SARS-CoV-2-induced neurological manifestations, including learning from HIV-1-induced neurological outcomes; presents the risk factors of COVID-19 in PLWH and the neurological impact of SARS-CoV-2/COVID-19 and the HIV-1/AIDS syndemic; and explores plausible mechanisms underlying neurological sequelae in SARS-CoV-2 infection of PLWH and people with HAND focusing on the NLRP3 inflammasome overactivation-associated neurotoxicity [38,39]. Additionally discussed are the challenges of COVID-19 on PLWH, including PASC and Long COVID incidences and possible ways to overcome the overwhelming influence of the SARS-CoV-2/HIV-1 syndemic.

## 2. Similarities and Differences between SARS-CoV-2 and HIV-1

SARS-CoV-2 and HIV-1 are natural RNA viruses transmitted to humans via a zoonotic transmission [40,41]. Despite differences, these viruses employ common molecular mechanisms during transmission and disease progression [3,41,42,43]. The similarities between SARS-CoV-2/COVID-19 and HIV-1/AIDS are described below and summarized in Table 1. The differences are presented in Table 2.

(a) Fear among the public is the most common attribute of both SARS-CoV-2 and HIV-1 viral infection. This public fear makes people psychologically ill, leading to stress and anxiety [42].
(b) Both SARS-CoV-2 and HIV-1 are enveloped viruses with single-stranded RNA as the genome.
(c) Both the genomes of SARS-CoV-2 and HIV-1 are prone to mutation, and the accumulation of mutations within the host under selection pressure results in the emergence of new variants. Furthermore, immunocompromised PLWH harbor SARS-CoV-2 for a longer time, providing ample time for mutant accumulation and resulting in the SARS-CoV-2 variant stemming [44].
(d) SARS-CoV-2 and HIV-1 have zoonotic origins and were transmitted to humans from animal reservoirs, HIV-1 from non-human primates (NHPs) and SARS-CoV-2 from bats.
(e) In respective natural reservoirs, SARS-CoV-2 and HIV-1 infections produce mild to no symptoms but incite disease upon human infection.
(f) One reason for the widespread COVID-19 and AIDS pandemics is the transmission of SARS-CoV-2 and HIV-1 via asymptomatically infected individuals.
(g) Lymphopenia as a result of drastic loss of CD4+T cells occurs due to HIV-1 and SARS-CoV-2 infection and is considered a prognostic marker [45,46,47]. There is a substantial drop in CD4+T cell counts in the acute phase of HIV-1 infection in contrast to the chronic phase, where a continued decline in CD4+T cells occurs and leads to AIDS. Lymphocytopenia is the hallmark of COVID-19 severity, but elevated levels of CD4+T and CD8+T cells were associated with milder disease conditions [45].
(h) SARS-CoV-2 and HIV-1 induce neutrophil extracellular traps (NETs) and cause NETosis, a neutrophil death mechanism. NETosis also may cause increased secretion of chemokines and cytokines, leading to increased inflammation.
(i) Both SARS-CoV-2 and HIV-1 induce higher proinflammatory cytokine secretion and inflammation.
(j) Increased serum levels of proinflammatory cytokines in SARS-CoV-2 and HIV-1 infected patients are considered to be biomarkers and are predictive variables associated with morbidity and mortality.
(k) Both these viruses, SARS-CoV-2 and HIV-1 infection, lead to immune dysregulation and are mediated by inflammasome activation. These viruses can activate NLRP3 inflammasome in different cells, including monocytes/macrophages and microglia. SARS-CoV-2-induced Microglial and macrophage NLRP3 inflammasome activation in the CNS results in neuroinflammation, causing myriad neurological manifestations.
(l) A portion of SARS-CoV-2 and HIV-1 infected individuals develop neurocognitive impairments.

## 3. HIV-1-Induced Neurological Manifestations

In addition to systemic infection and immune dysregulation, HIV-1 infects the CNS and causes neurological manifestations. CNS infection by HIV-1 was recognized early in the HIV-1 epidemic [30,48,49]. The presence of HIV-1 in the CSF and brain tissues within days of primary systemic infection indicates HIV-1 neuroinvasion early during the course of infection [49,50,51,52,53]. Despite controversy regarding how exactly HIV-1 enters the brain, the Trojan horse mechanism of HIV-1 infection of monocytes and lymphocyte trafficking and penetrating the blood–brain barrier (BBB) is the most convincing in terms of CNS infection [54,55,56]. Thus, HIV-1 neuroinvasion occurs primarily via immune cell trafficking across the BBB and subsequent dissemination by infection of perivascular macrophages/monocytes and lymphocytes [25,50,53,57,58]. HIV-1 does not infect neurons. Neuronal injury is believed to be mediated by HIV-associated neuroinflammation and neurotoxic viral proteins (gp120, Tat, Nef and Vpr). The productive viral replication produces a prolonged inflammatory environment resulting in sustained neuroinflammation [59,60], leading to the pathogenesis of HAND [59,60]. HAND pathogenesis and progression may also be the consequence of the reactivation of HIV-1 reservoirs in the brain [61,62,63]. HAND can be categorized by neuropsychological tests and/or assessment of functional status into three categories (i) Asymptomatic neurocognitive impairment (ANI), (ii) Mild neurocognitive disorder (MND) and (iii) HIV-1-associated dementia (HAD) [59,60,64,65]. 

Prior to cART, HAD was found in 25% of HIV-1-infected individuals [30]. Although the incidence of HAD has declined significantly in the cART era, more than 50% of the PLWH exhibit a milder form of HAND [50]. The clinical manifestations of HAND are memory impairment, attention disruption, poor judgment and challenges in multitasking [65,66]. The severity of HAND worsens with disease progression due to motor dysfunction, resulting in coordination disruption, executive dysfunction and end-stage dementia [65,66]. Whether HAND is a legacy effect of residual viral replication in the CNS after cART or due to the systemic inflammation mediated by glial cells is still unresolved and warrants dedicated investigation. The lessons learned from comprehensive research on HIV-1-induced neurological manifestations may provide researchers with the following pathways for studying SARS-CoV-2-induced CNS damage: (a) direct infection of the CNS glial cells and macrophages, (b) direct neurotoxic effect of viral proteins on resident cells and (c) hyperactivated immune cell trafficking into the brain, the neuroimmune axis [67]. An investigation of the above-mentioned pathways may enhance our understanding of the potential impact of SARS-CoV-2 on the brain, especially on PLWH and PLWH with HAND in SARS-CoV-2/HIV-1 syndemic. Nevertheless, the acquired knowledge from HIV-1 research on the CNS may provide insight into the impact of SARS-CoV-2-induced neurological manifestations.

## 4. Impact of SARS-CoV-2 on the CNS

The newly emerged novel coronavirus SARS-CoV-2 is a spillover from bats and is primarily a respiratory pathogen [68,69,70,71]. The SARS-CoV-2 infection proliferates to other organ systems in infected individuals, such as the renal, cardiovascular, and nervous systems [24,26,27,68,72]. Recent postmortem studies of COVID-19 victims have detected SARS-CoV-2 RNAs in the brain tissues and cerebrospinal fluid (CSF) [73,74,75]. Neuroinvasion of SARS-CoV-2 was revealed by immunostaining of viral nucleocapsid and/or spike protein [33,76]. SARS-CoV-2 invasion into the CNS was observed in COVID-19 animal models (NHPs, mice and hamsters) and the autopsy of COVID-19 patients [33,35,77]. Further, studies on brain organoids also demonstrated the neuroinvasion of SARS-CoV-2, like other coronaviruses, such as SARS-CoV and MERS [78,79,80]. Worldwide studies have revealed that SARS-CoV-2 enters the CNS by crossing the BBB or/and via retrograde neuronal transfer through the olfactory nerves. Despite evidence of SARS-CoV-2 neuroinvasion, how exactly this virus enters the CNS and its precise timing warrant further investigation. SARS-CoV-2 also causes neurological sequalae without CNS infection, implying indirect viral effects on the brain, such as abortive infection and/or viral proteins in the CNS. Nonetheless, understanding the influence of SARS-CoV-2 and viral proteins on the brain and resultant neurological consequences is imperative.

**Figure 1:**
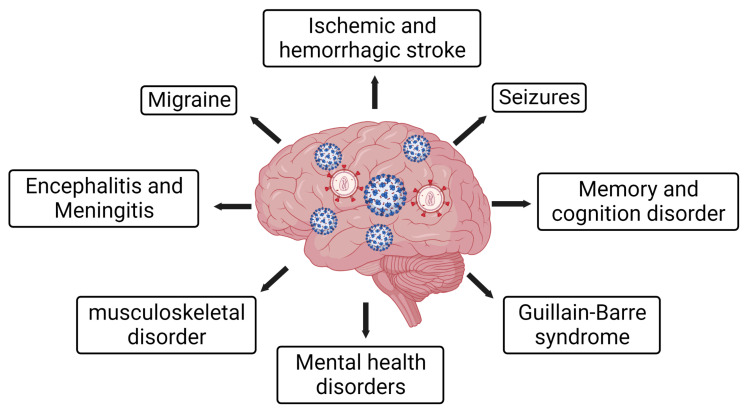
Neurological disorders associated with SARS-CoV-2/COVID-19 and HIV-1/AIDS syndemic. COVID-19 has a broad impact on neurological manifestations and specifically on HIV-1/ADIS patients. COVID-19 may worsen neurological disorders, including ischemic/hemorrhagic stroke, encephalitis, meningitis, seizures, migraine, mental health disorders, memory and cognition disorders, musculoskeletal disorders and Guillain–Barre syndrome.

Emerging neurological symptoms reported in COVID-19 patients include headache, seizures, stroke, encephalopathies, altered mental status, and many more (Figure 1). SARS-CoV-2 was identified as a primary respiratory pathogen early in the pandemic, and it has been confirmed to be a neuropathogenic virus [26,81,82]. There is evidence indicating a restricted SARS-CoV-2 infection in the CNS [83]. The lower level of SARS-CoV-2 viremia than a typical viral brain infection suggests an alternate mode of CNS infection rather than crossing BBB [84]. In parallel with the viral load level in the blood, the level of viral load in the CSF was also low, indicating an unlikely possibility that productive viral infection/replication in the CNS is solely responsible for COVID-19-induced neurological outcomes [84]. Since angiotensin-converting enzyme 2 (ACE2) and transmembrane protease serine 2 (TMPRSS2) are expressed in vascular cells such as pericytes and immune cells (monocytes/macrophages, microglia) [84,85] but not neurons, and neuronal injury could be an indirect effect of viral CNS infection. Nevertheless, the possible ways that SARS-CoV-2 implies COVID-19-induced neurological outcomes could be direct infection into the CNS, viral neurotoxic proteins and the neuroimmune axis.

There is agreement regarding SARS-CoV-2 entry into the CNS, and the nose is considered to be the front door, while the retina is the window [84,86]. After infection, SARS-CoV-2 replicates in the upper respiratory tract. Thus, most detection methods for COVID-19 use nasopharyngeal swab samples to detect the presence of the virus [87,88]. The entry of SARS-CoV and SARS-CoV-2 into the human host occurs by binding of viral S-protein to host cell receptor ACE2 and TMPRSS2 priming of S-protein [89]. Previously SARS-CoV intranasal infection was demonstrated using mice expressing human ACE2 [90,91]. Similar to bronchial transitory secretory lung cells, it was found that some neuronal and glial cells in the CNS also have ACE2 expression [82,85,92]. Moreover, under physiological conditions, some olfactory mucosal non-neuronal cells also express the ACE2 receptor [93]. Further, using regional mapping, the presence of a higher level of SARS-CoV-2 RNA was shown in the olfactory mucosa. Due to the proximity of neurons and nerve fibers in the olfactory region and the signs of alteration in taste and smell perceptions, it was suggested that the neural–mucosal interface is a promising port of SARS-CoV-2 entry into the CNS [86]. The high prevalence of S-protein in the olfactory mucosa also indicates SARS-CoV-2 neuroinvasion via the transmucosal route along the olfactory tract [86]. Studies using autopsy samples from COVID-19 victims revealed SARS-CoV-2 replication in the olfactory bulb and hypothalamus, suggesting the olfactory route of CNS invasion [33,94,95]. In another study carried out on the K18-hACE2, a mouse model of COVID-19 intranasal infection, the authors detected SARS-CoV-2 brain infection and the upregulation of neuroinflammatory markers [96]. It was in that study that a productive infection of SARS-CoV-2 occurred in the nasal turbinate cells, olfactory bulb and eyes, supporting the olfactory route of the CNS infection [96]. This route of CNS infection was further supported by experimental results that SARS-CoV-2 RNA was detected in blood and CSF one day after intranasal infection, further stipulates lymphatic/hematogenous virus spread via BBB endothelial cell infection to disseminate into the CNS [97,98]. A recent study has reported CNS damage and disruption in BBB integrity in the acute phase of COVID-19 patients with severe neurological symptoms [99]. In this study, authors have shown increased plasma neuro–axonal damage specific biomarker neurofilament light chain (NfL) levels in COVID-19 patients, specifically those with ARDS [99]. Further, matrix metalloproteinases (MMPs) are zinc-dependent enzymes and mediators of neuroinflammatory processes regulating BBB integrity and were found to be upregulated in COVID-19 patients. Additionally, in neuro-COVID-19 patients, increased levels of both plasma and CSF NfL and MMP-2 (a form of MMPs) were found in ARDS compared to non-ARDS groups [99]. Thus, this study confirms the CNS damage and breach in BBB integrity in COVID-19, which may lead to Long COVID or PASC.

SARS-CoV-2 neurotoxic proteins play a crucial role in neurological outcomes. In addition to productive brain infection of SARS-CoV-2, an abortive SARS-CoV-2 infection can elicit inflammatory responses in brain cells [67]. Additionally, SARS-CoV-2 may be trapped inside lysosomes of microglia, astrocyte and macrophage and viral proteins may stimulate Toll-like receptors (TLRs) to initiate an inflammatory cascade [100]. Thus, SARS-CoV-2 proteins may modulate the neuroimmune milieu and be responsible for COVID-19-induced neurological outcomes. Like other viral proteins, SARS-CoV-2 spike protein in the CNS induces neuroinflammation by promoting the release of neurotoxic cytokine via the microglial inflammasome activation [35]. Thus, in addition to other mechanisms, inflammasome activation by SARS-CoV-2, and viral proteins play a substantial role in the neuroinflammation-induced COVID-19-associated neurological consequences. How SARS-CoV-2 proteins induce neuroinflammation and neurodegeneration via NLRP3 inflammasome activation is discussed in the mechanism section of this review.

The neuroimmune axis is another potential way to modulate COVID-19-induced neurological syndrome. The brain is an immune-privileged site, and immune cells traffic to the CNS in the absence of infection or BBB breakdown at a slower rate and less in number than other parts of the body [101]. Brain infection produces inflammatory responses and enhances immune cells trafficking into the brain. Such an immune response is regulated to balance the inflammatory response to contain the viral infection. As a standard belief, the Trojan horse theory for viral dissemination to various organs is widely accepted in which migratory immune cells can infiltrate diverse tissues [102,103]. Thus, the Trojan horse theory explains how brain infection occurs, where brain-infiltrated monocytes differentiate into resident tissue macrophages and spread the virus [102,103]. A noteworthy point for HIV-1 infection is that not monocytes but monocyte-derived macrophages support productive HIV-1 brain infection [104]. SARS-CoV was previously known to infect brain macrophages/microglia (CD68+) and T cells (CD3+), as evident from histochemical analysis of human brain autopsy samples [105]. It has recently been reported that SARS-CoV-2 infects monocytes, but this infection was abortive. The abortive infection induces inflammation by inflammasome activation-mediated proinflammatory cytokine production [106]. Moreover, the abortive infection of monocytes may introduce viral proteins into the CNS, resulting in neuroinflammation by microglial NLRP3 inflammasome activation. 

Additionally, COVID-19 survivors have a broad spectrum of neurological symptoms, including headache, impaired concentration, fatigue, hyposmia and myalgia long after infection as a constellation referred to as the PASC or Long COVID [107,108,109]. Symptoms of Long COVID also include fatigue, encephalitis, cerebrovascular disease, mental health disorder, cognitive problem, neuropathies and muscular disorders (Figure 1) [108]. The Long COVID symptoms were more severe in older persons with comorbidities than in young, healthy adults [110]. More severe symptoms were also observed in people with Long COVID who have a preexisting neurological syndrome or developed because of COVID-19, such as stroke or encephalitis [111]. Considering these factors, it is worth mentioning that conditions with PLWH, especially with HAND, may lead to double jeopardy [112]. The following section delineates the syndemic effects of SARS-CoV-2/HIV-1 and their impact on neurological manifestations. 

## 5. Impact of COVID-19 on PLWH

In addition to its devastating impact on the world population, the COVID-19 pandemic leads to double jeopardy for PLWH, especially those who have progressed to HAND [112]. Despite the contradictory views regarding the enhanced risk of SARS-CoV-2 infection in PLWH [13], it has been confirmed that the PLWH are at paramount risk of mortality and morbidity compared to HIV-negative people [113,114,115]. In PLWH, comorbidity, age, sex and ethnicity are detrimental risk factors for SARS-CoV-2 infection and COVID-19 severity that may lead to increased mortality and morbidity [113,114,115]. 

### 5.1. Risk Factors Associated with COVID-19 in PLWH

In the current cART era, PLWH with controlled viremia are living an almost normal daily life with similar life expectancies comparable to HIV-1-negative people [50,65,116,117,118,119,120]. However, the restricted cART penetration into the CNS through the BBB resulted in persistent low-grade viral replication in the brain. The residual HIV-1 replication and viral neurotoxic proteins mediated chronic immune activation and persistent inflammation leading to the development of HAND. As most PLWH are older people, who are often burdened with comorbidities, including autoimmune disease as risk factors, further boosting SARS-CoV-2 infection and COVID-19 severity. It was found that immune system dysregulation imparts a higher risk of COVID-19 severity in PLWH than in non-PLWH [20,121]. Intriguingly, the risk factors influencing SARS-CoV-2 infection and COVID-19 severity are complex and determined by target cell ACE2/TMPRSS2 expression and environmental exposure [122]. Listed below are the diverse risk factors associated with SARS-CoV-2 infection and COVID-19-imposed exacerbation of HAND,

i. Age: It is well known that older people are more susceptible to the COVID-19 pandemic. At the beginning of the pandemic, biological age was recognized as a significant risk factor. Several studies revealed that older people are more likely to become infected with SARS-CoV-2 and COVID-19-related hospitalization and mortality. According to the Centers for Disease Control and Prevention (CDC), people aged 85 or older have a 13-fold higher risk of hospitalization and 630-fold death than those aged 18–29. However, people between 50 and 85 are at a 4–8-fold higher risk of hospitalization and 30–220-fold of death [122,123]. As a result of stringent cART regiments, half of PLWH in the United States are 50 or older [118,119,124]. These older PLWH with several age-related comorbidities and compromised immune systems are at increased risk of COVID-19 complications. They usually experience physical and cognitive impairments, chronic immune activation and multimorbidity even without COVID-19 at ages younger than HIV-1 negative people [125,126,127,128,129]. Thus, aging and age-associated comorbidity make PLWH succumb to SARS-CoV-2 infection and COVID-19 severity [130]. Since COVID-19 also causes neurological syndromes, SARS-CoV-2 may imply a cumulative effect on preexisting HAND or may lead to the development of HAND. Nonetheless, investigating the impact of COVID-19 on aged PLWH with HAND is imperative in order to mitigate the devastating impact of the syndemic.
ii. Sex: There are gender biases recorded for SARS-CoV-2 infection and COVID-19-mediated disease severity. It was found that SARS-CoV-2-infected men aged between 40 and 70 years proceed to COVID-19 severity with double the risk of mortality than the same age group of women [131,132,133,134]. The sex-biasedness in the risk of SARS-CoV-2 infection and COVID-19-induced disease severity is most likely due to sex-based differential immune responses and immunomodulatory effectors such as sex hormones and sex-specific comorbidity [135]. The sex-based biases are possibly due to male-induced nonclassical monocytes and increased cytokines (IL-8 and IL-18) production in contrast to women-induced robust CD8+T cell response [136]. Additionally, lesser vulnerability and COVID-19 severity in women may be associated with enhanced neutrophil activity and increased type I interferon (IFN-I), generating robust innate immune response via TLRs [137].
  Additionally, in males, the ACE2 and TMPRSS2 responsible for SARS-CoV-2 infection and pathogenesis were elevated compared to females [89,138,139]. Both ACE2 and TMPRSS2 are androgen-responsive [140,141,142,143]. Men with lower testosterone levels were found to be associated with severe COVID-19 outcomes [140,141,142,143]. The lower testosterone levels in men were also linked to higher proinflammatory cytokine [144]. Thus, androgen deficiency and testosterone dysregulation could modulate ACE2 and TMPRSS2 expression, further influencing COVID-19 outcomes in PLWH [145]. In contrast, the impact of COVID-19 in PLWH was the opposite regarding sex bias, with increased morbidity and mortality recorded in women than in men [146,147]. Such a discrepancy could be due to stronger immune activation and increased inflammatory markers in women, despite similar viral suppression among men and women under strict cART regimens [148]. Although these initial studies have concluded the opposite incidence of SARS-CoV-2 infection and COVID-19 outcomes in PLWH, the sex-biased differential impact warrants further investigation and critical interpretation.
iii. Comorbidities: As mentioned above, older people are at higher risk of COVID-19 severity; this may be due to the presence of comorbidities in people of this age group. PLWH are often with several comorbidities, including dysregulated immunity, chronic diseases in the lung, kidney and liver, and obesity, diabetes, hypertension, hyperlipidemia, cardiovascular disease and other health issues, which may exacerbate the COVID-19 severity [21,22,149,150,151,152,153]. PLWH with multiple comorbidities are victims of disease severity and possess an increased chance of mortality [154,155]. However, studies have revealed that PLWH adhering to cART, with adequate CD4+T cell count, viral suppression and without comorbidities do not have a higher risk of COVID-19 severity and mortality than non-PLWH [156]. Future studies are warranted to delineate how these comorbidities affect COVID-19 severity and mortality in PLWH and PLWH with impaired neurocognitive function.
iv. Immunity: The role of the immune system is to protect us from infections and diseases. Any alteration or dysregulation in the immune system makes us vulnerable to infection and severe disease outcomes. People with compromised immunity are at higher risk of SARS-CoV-2 infection and COVID-19 severity. In PLWH, the immune system is dysregulated despite the cART regimen due to a lack of immune reconstitution or loss of immunological memory [157,158,159]. In a recent study, the cART regimen exhibited potential protective effects on the incidence and severity of COVID-19 in PLWH [160]. However, after clinical trials of several antiretrovirals used to treat HIV-1, only partial protection was observed, and more clinical trials may provide us with a better understanding of using antiretrovirals to treat SARS-CoV-2 infection [161]. Another study on PLWH showed that neither HIV-1 plasma viral load nor CD4+T cell count at the time of diagnosis determined COVID-19 outcomes [121], despite SARS-CoV-2 and HIV-1 infection-mediated T cell lymphopenia [46]. On the contrary, recent studies presented on CROI 2022 reported the worst COVID-19 clinical outcomes in PLWH with recent CD4+T cell counts of <200. More dedicated meta-analysis is required to evaluate the effects of antivirals on SARS-CoV-2 infection and COVID-19 severity. Viruses can inhibit T cell receptor signaling and immune response, resulting in immune system dysregulation and imposing severe outcomes of COVID-19 in PLWH. Furthermore, the emergence of SARS-CoV-2 variants make PLWH who are immunocompromised more susceptible to accumulating mutations during viral replication. These immunocompromised individuals may harbor viruses for a longer time, providing an opportunity for variant emergence. HIV may “trigger” the emergence of SARS-CoV-2 variants reported by a study carried out in South Africa because of the high number of PLWH in this region [44,162].
v. Socioeconomic inequalities: In this world, millions of people suffer from the risk of the social determinants of health inequalities, including the economic conditions for affordable healthcare due to poverty [163]. PLWH are often with socioeconomic burden due to health conditions and loss of income sources. Poor people with financial burdens are unhealthy due to poor diet and inability to afford appropriate healthcare; thus, the people in this group are more vulnerable to infection and disease progression to severity. The living hygiene condition deteriorates with food insecurity and the absence of proper care. Further, the economic uncertainty is disproportionate and evident in some communities, such as the black and Hispanic populations, and people in these communities represent most sufferers of these conditions. There are several worldwide studies on determination of socioeconomic factors that affect disease progression and related mortality. It is believed that socioeconomic burden disproportionately impacts the risk of SARS-CoV-2 infection and disease severity. Thus, poor people with socioeconomic disadvantage are in the high-risk category for SARS-CoV-2 infection and disease severity due to their poor health conditions and immunocompromised status.
vi. Substance abuse: Early in the COVID-19 pandemic, a steep rise in substance abuse, drug overdose and mental health challenges was reported [164]. Among PLWH, drug abuse is common, further burdening COVID-19 severity and mortality as COVID-19 imposed social isolation resulting in mental health deterioration and strained PLWH toward substance use disorders (SUDs) [165]. People with SUD (PWSUD) might have greater exposure and health challenges leading to higher susceptibility to SARS-CoV-2 infection [166]. PWSUD are poor in adhering to social distancing and following COVID-19 guidelines; this may be due to sharing syringes for substance use, especially in large congregate settings [167,168,169]. People with previously diagnosed SUDs have an 8-fold higher possibility of SARS-CoV-2 infection than people without SUD [170]. Thus, it is evident that PWSUD have a higher possibility of SARS-CoV-2 infection and COVID-19 severity. Different SUD types also determine exposure and illness, such as people with opioid use disorders (OUD) and cannabis use disorder (CAUD), who are at 10.2% and 5.3% higher likelihood of SARS-CoV-2 infection and COVID-19 outcomes [170]. In addition, alcohol use disorder (AUD) and Methamphetamine (MA) use disorder also increased due to the lockdown and stay home policy. There is an increased incidence of COVID-19 diagnosis, hospitalization and fatal outcomes in PWSUD compared to people without SUD [170,171]. Some PWSUD may have dysregulated immune responses and comorbidities, including HIV-1 infection, which can further exacerbate COVID-19-mediated complications [172]. There are reports of psychiatric association with SUD and increased incidence of COVID-19-related hospitalization and extended hospital stay [173]. Overall, PWSUD have higher vulnerability for SARS-CoV-2 infection and COVID-19 outcomes.

Among PWSUD, MA abusers were linked to immune dysfunction in men who have sex with men (MSM) [174]. In MSM, immune dysfunction exacerbates the transmission of HIV-1 and other sexually transmitted infections (STIs) compared to heterosexual men [174]. A study reported that MA abuse was found to cause neuronal mental health issues in MSM and develops neurocognitive impairments [175]. Frequent MA abuse in MSM provokes avoidance of COVID-19 preventive measures, culminating in an enhanced risk of SARS-CoV-2 infection [176]. Therefore, PLWH with drug abuse are at paramount risk of SARS-CoV-2 infection and COVID-19-induced severe outcomes. Managing or controlling MA abuse among PWSUD with appropriate measures and treatment may provide proper ways to avoid the disease and COVID-19-associated severity.

### 5.2. SARS-CoV-2 Infection and COVID-19 Severity in PLWH

According to a report from the joint United Nations Programme on HIV/AIDS (UNAIDS), PLWH are at higher risk of contracting SARS-CoV-2 infection but have less access to the COVID-19 vaccine [17]. Despite several studies, there is no clear consensus whether PLWH are at higher risk of contracting COVID-19 or protected from SARS-CoV-2 infection due to antiretroviral therapy against HIV-1 [177]. It is contemplated that PLWH are more vulnerable to SARS-CoV-2 infection [177]. Several cohort studies on PLWH have demonstrated that PLWH are among the high-risk group for contracting SARS-CoV-2 infection [16,178,179,180]. PLWH are more likely to be infected with SARS-CoV-2 due to immunocompromised status owing to HIV-1 infection-induced dysregulated immune response/immune suppression and underlying comorbidities [130,181]. Moreover, there was complex immune dysregulation observed with higher levels of IFNα/β and T-cell activation in coinfected people than in healthy controls [182,183]. Low CD4+T counts with superimposed lymphopenia may have an adverse effect on PLWH due to COVID-19 and worsen the disease outcomes [183]. However, more studies are needed to explain no significant increase in mortality in PLWH due to COVID-19 reported in some studies [184,185,186]. It has been demonstrated that COVID-19 patients admitted to ICU were found to have significantly lower CD8+T and CD4+T counts, and these parameters were negatively correlated with survival [187]. In addition, dysregulated immunity and age-related comorbidities exacerbate COVID-19 outcomes in PLWH and increase mortality and morbidity.

It has been noticed that PLWH are more vulnerable to COVID-19, at least in part due to the disproportionate distribution of PLWH in the world, with higher numbers in the African subcontinent superimposed by lower COVID-19 vaccine availability [177]. Additionally, COVID-19-related restrictions in social activities, including gathering and traveling, etc., have an unprecedented disruption in daily lives, HIV-1 testing and healthcare delivery [188,189,190]. There have been tremendous changes to global healthcare systems to prioritize the fight against the COVID-19 pandemic [191,192]. These changes have imposed adverse consequences on PLWH, and even they have difficulty in refilling antiretrovirals or availing cART [191,192]. This disruption in healthcare services may severely affect PLWH as the viral load rebounds and increase the chance of other opportunistic infections, including an increased risk of SARS-CoV-2 infection [193]. It was noticed that PLWH were in a compromised state of psychological and emotional well-being in addition to physical well-being during the COVID-19 pandemic due to difficulty in obtaining cART and proper care/support from society and family [12,192]. This psychosocial issue of PLWH must be immediately addressed to avoid severe adverse outcomes [12]. 

### 5.3. COVID-19 Imposed Neurological Outcomes on PLWH 

The COVID-19 pandemic impacts the global population, and numerous studies have reported various neurological implications associated with the disease [37,194,195]. SARS-CoV-2-mediated neuroinflammation is associated with neurological manifestations observed clinically. During the COVID-19 and HIV syndemic, a large global population (38.4 million) is HIV-1-infected, with the maximum burden on the African subcontinent (25.7 million) [196,197]. Thus, monitoring COVID-19-induced neurological outcomes on PLWH and neurocognitively impaired PLWH is contextual. 

SARS-CoV-2 induces immune dysregulation, causing a surge in proinflammatory cytokine secretion called cytokine storm syndrome (CSS). These excessively released cytokines damage the BBB and infiltrate SARS-CoV-2 infected cells into the CNS, causing increased neuroinflammation [198,199]. SARS-CoV-2-mediated immune dysregulation occurs due to the induction of oxidative stress and proinflammatory genes, resulting in inflammatory stress and cytokine storm [200,201,202]. In the CNS, SARS-CoV-2 causes the excessive release of proinflammatory cytokines (IL-1β and IL-6) by glial cells, producing CSS-like conditions [203]. Further, SARS-CoV-2 activates CD4^+^ T cells in the CNS and induces macrophages to secrete IL-6 by producing GM-CSF [204]. Though SARS-CoV-2 is in its infancy, studies on other coronaviruses have confirmed that SARS-CoV-2 can augment CNS inflammation [37].

PLWH, especially those with HAND, are characterized by altered neuroinflammation with dysregulated inflammatory markers [205,206,207]. The immune system in HAND patients is compromised, with dysregulated peripheral and CSF IL-6 levels [208,209,210]. In advanced stages of HIV-1 infection, the serum and CSF IL-6 and GM-CSF levels were elevated, demonstrating dysregulated immune responses at systemic as well as at CNS levels [208,209,211]. In addition, the dysregulation of alternative markers such as CRP and VGEF in HAND and an increased level of VGEF were associated with the HAND severity [210,212]. In COVID-19, CSS was found to be a common phenomenon, and IL-6 was a predominant cytokine [213,214,215]. The onset of CSS dysregulates the IL-6 pathway and plays a pivotal role in COVID-19 pathophysiology [213,214,215]. An elevated level of IL-6 in COVID-19 was correlated with disease severity and mortality and used as a tool for disease prognosis and clinical profile [215]. It was postulated that SARS-CoV-2-induced higher IL-6 in the CSF could negatively impact neuronal health in PLWH. Both HIV-1 and SARS-CoV-2 can cause the production of IL-6, resulting in a synergistic effect on neuroinflammation. The synergistic impact may promote and prolong neuroinflammation. It has been reported that CSS causes compromised neurological presentations, such as altered levels of consciousness with increased levels of IL-6 and CRP [216]. Dysregulated levels of CRP and VEGF were also reported in patients with COVID-19 and were used as biomarkers for disease prognosis [94,217,218]. These parameters were found to be altered in patients with HAND as well [210,212]. It is conceivable that a cumulative effect may amplify hyperinflammation and BBB dysfunction in coinfected individuals.

It is worth emphasizing that a more significant threat for PLWH could exist as immune dysregulation induced by SARS-CoV-2 infection may lead to the reactivation of HIV-1 viral reservoirs in the CNS. Thus, the SARS-CoV-2-induced HIV-1 surge may expand the reservoir size and inflammatory profile, enforce accelerated cognitive disorder and worsen HAND. Such viral reservoir reactivation-induced diseases were also found in viral infections other than HIV-1 [219,220]. The viral reservoir can also be activated by mRNA vaccine against COVID-19, the BNT162b2 [219,220]. Because of this, the COVID-19 vaccine-induced HIV-1-reservoir reactivation may have unforeseen consequences and generate the concern of vaccine hesitancy among PLWH. Therefore, it is time to critically evaluate the long-term safety of the SARS-CoV-2 vaccines in PLWH. 

In addition to the aberrant neuroimmune response, widespread vascular dysfunction has been reported in COVID-19 [26]. The COVID-19-induced vascular dysfunctions include cerebrovascular events and contribute to neurological complications [26]. COVID-19-induced system-wide vascular dysfunction predicts disease severity and contributes to organ failure [221]. These cerebrovascular events and brain tissue damage may adversely impact the CNS and lead to severe neurological outcomes. A recent study using a high magnetic resonance of brain tissue found that microvascular damage was associated with COVID-19 [222]. This study further indicated that the microscopic brain damage may underlie COVID-19-associated neurological manifestations [222]. In some cases, the damage may lead to short-term post-infection complications. However, in other cases, such COVID-19-induced vascular brain damage may lead to a lingering syndrome known as PASC or Long COVID [26,109,222]. COVID-19-induced PASC or Long COVID appears with persistent myriad neurological and psychiatric illnesses [26,109,222]. The PASC symptoms include difficulty in concertation, loss of memory creating brain fog with confusion, headache, intractable fatigue and inability to accomplish daily activities, delirium and many others [223]. To characterize the long-term neurological outcomes of COVID-19, a cohort study was conducted post-12 months following acute SARS-CoV-2 infection, and the results showed an array of neurologic incidences [108]. The COVID-19-induced neurologic sequelae include ischemic and hemorrhagic stroke, memory and cognition disorder, migraine, seizures, sensory and mental health disorder, musculoskeletal and movement disorder, encephalitis and Guillain–Barre syndrome (Figure 1) [108]. Thus, it is evident from the study regarding the enhanced risk of long-term neurologic syndrome in people who succumbed to COVID-19. Therefore, COVID-19 introduces a cumulative threat to PLWH and people with HAND, with a significant risk incidence for PASC or Long COVID. To date, there is a paucity of studies on PASC in PLWH and HAND patients. Therefore, it is urgent to study the HIV-1/SARS-CoV-2 syndemic in order to gain insight into the adverse impact of COVID-19 on PLWH. An attempt to characterize PASC in PLWH infected with SARS-CoV-2 revealed that a dysregulated adaptive immune response was responsible for these long-term effects [224]. An analysis of PLWH recovering from SARS-CoV-2 infection concluded that the high levels of inflammation and dysregulated inflammatory markers contributed to PASC [224]. COVID-19 severity and the requirement for emergency hospitalization in PLWH were due to PASC [225,226]. 

PASC or Long COVID is partially due to ongoing or residual immune activation and inflammation as the consequence of SARS-CoV-2 infection [224,227]. As PLWH under the cART regimen is immunocompromised with persistent chronic immune activation and inflammation [228,229,230], additional immune perturbation induced by SARS-CoV-2 sequelae could enhance the severity of PASC. Long-COVID could be further exacerbated in PLWH with autoimmune disease, comorbidities, microvascular dysfunction, localized tissue inflammation, and reinfection or opportunistic infections [231,232,233,234]. Therefore, PASC or Long-COVID may be debilitating and life-threatening in PLWH, especially those with cognitive impairment. However, a recent study reported complete protection against CNS infection and brain damage by SARS-CoV-2 in animals vaccinated with modified vaccinia virus Ankara expressing SARS-CoV-2 S-protein (MVA-CoV-2-S) [235]. Hence, there is hope that vaccines against COVID-19 may protect from neurological syndromes. Thus, people with HAND may benefit if vaccinated and protected prior to SARS-CoV-2 infection or at least from COVID-19-mediated ARDS. It is worth noting here a recent finding that observed higher levels of plasma and CSF NfL and MMP-2 in the acute phase of COVID-19 with ARDS compared to non-ARDS [99]. NfL and MMP-2 are biomarkers of CNS damage and BBB integrity, and higher levels indicate the stimulation of neuroinflammatory processes that may worsen preexisting HAND or may initiate HAND in PLWH. Therefore, as mentioned above, COVID-19 vaccination may reduce the chances of ARDS, and patients may be protected from CNS damage and BBB disruption. Future studies on animal models and humans using the vaccine against SARS-CoV-2 may provide better insight into CNS protection. Given that COVID-19 vaccination has such an impact on CNS infection, PLWH are strongly suggested to be vaccinated without hesitancy to protect from adverse neurological outcomes. Thus, vaccine awareness programs must be encouraged worldwide in order to obtain herd immunity against SARS-CoV-2.

## 6. Mechanisms of COVID-19-Induced Neurological Manifestations in PLWH

The COVID-19 pandemic enormously impacts the world population, specifically people with comorbidities [23]. SARS-CoV-2/COVID-19 not only affects the respiratory and immune systems but also attacks the nervous system, producing neurological consequences. How SARS-CoV-2/COVID-19 impacts PLWH is not fully understood. It is well-known that HIV-1 infection induces a hyperactive immune response causing systemic inflammation and neuroinflammation. Introducing another virus on top of HIV-1 may further dysregulate the immune system. Thus SARS-CoV-2/HIV-1 coinfection may result in cumulative enhancement of neuroinflammation, leading to the exacerbation of neurogenerative conditions and HAND via various mechanisms. 

### 6.1. Inflammasome Activation

The immune system is continuously working with high priority to protect us from infectious diseases and injury. As a first line of defense, the innate immune system plays a pivotal role in saving us fromCOVID-19. The activation of innate immune sensors by pathogen-associated molecular patterns (PAMPs) and/or danger-associated molecular patterns (DAMPs) provides defense against pathogens or host-assaulted injury. Stringent regulation of immune responses is a fundamental requirement to keep us healthy, and their dysregulation may result in serious outcomes. Among these protective sensors, inflammasomes are prominent in innate immune regulation for viral infections. The NLRP3 inflammasome is one of the most studied and well-explored/characterized innate immune complexes involved in COVID-19. SARS-CoV-2 modulates the NLRP3 inflammasome, especially in COVID-19/AIDS syndemic [236,237,238]. SARS-CoV-2 and HIV-1 activate the inflammasome in many organs and cells, including lungs, monocytes/macrophages and microglia [239,240,241,242,243,244,245,246]. To date, few studies have investigated NLRP3 in SARS-CoV-2-infected PLWH (SARS-CoV-2/HIV-1 syndemic). Mechanistically, SARS-CoV-2-induced NLRP3 inflammasome activation imposes neurological syndromes, PASC/Long COVID, and exacerbates neurocognitive impairments in PLWH. 

**Figure 2:**
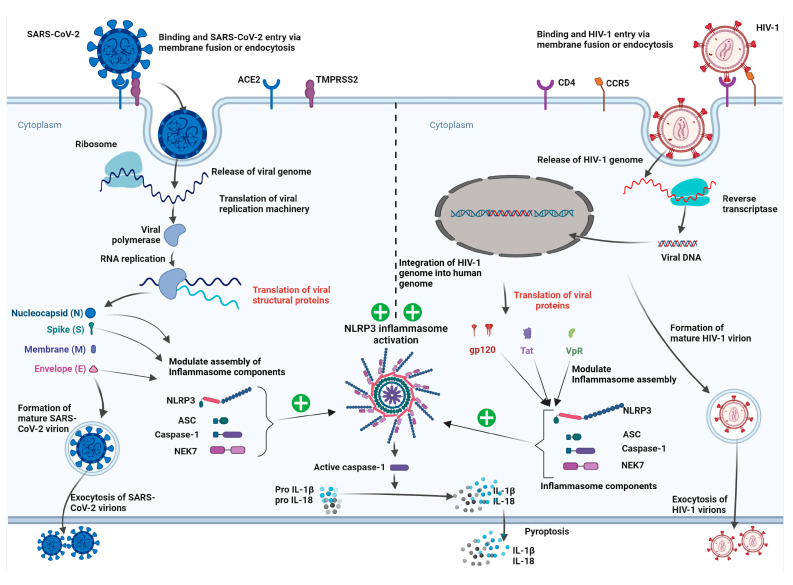
Mechanisms underlying SARS-CoV-2/HIV-1-associated neurological disorders. Both these viruses can activate microglial NLRP3 inflammasome and cause excessive secretion of proinflammatory cytokines. Thus, these viruses may have a cumulative effect on proinflammatory cytokine secretion, which may have profound neurodegenerative and neurotoxic effects and may worsen neurological disorders.

In Figure 2, we attempt to extrapolate and hypothesize the plausible mechanisms involved with the neurological sequelae resulting from SARS-CoV-2/HIV-1 coinfection. HIV-1 infects the CNS early during infection and replicates in the macrophage and microglia [247]. The discovery of the CNS as an HIV-1 reservoir where the viral genome integrates into the microglial/astrocyte genome has a profound consequence regarding reservoir reactivation [61]. In addition to systemic inflammation, HIV-1 infection and viral proteins cumulatively prolong the inflammatory environment in the CNS [245,246,248,249,250]. Despite cART, the chronic neuroinflammatory environment is prevalent due to low-grade viral replication and immune dysregulation in PLWH. In parallel, SARS-CoV-2 infection also causes immune dysregulation and widespread cytokine storm [251,252]. In addition to this systemic perturbation, SARS-CoV-2 also induces neuroinflammation [35,253,254]. SARS-CoV-2-mediated neuroinflammation is the direct effect of viral neuroinvasion and substandard effect by shaded protein-induced inflammasome activation and the BBB-disruption [35,253,254]. SARS-CoV-2 and HIV-1 generate an inflammatory environment in the CNS by activating the NLRP3 inflammasome in microglia and monocytes/macrophages [35,239,245]. The NLRP3 inflammasome activation increases the production of neurotoxic proinflammatory cytokines [35,239,245]. In the SARS-CoV-2/HIV-1 syndemic, both viruses cumulatively activate the NLRP3 inflammasome, and such a synergistic effect may lead to a prolonged chronic proinflammatory and immune-dysregulated milieu. Thus, synergistic neural hyperinflammation induced by both viruses may further exacerbate preexisting HAND and/or initiate the HAND phenotype in PLWH (Figure 2). Indeed, the overactivation of NLRP3 inflammasome has been observed in aged people [255]. The overactivation of NLRP3 inflammasome in the macrophages of aged people is attributed to an increased mitochondrial reactive oxygen species (mtROS), mitochondrial DNA (mtDNA) and impaired mitochondrial functioning [255]. The inflammasome hyperactivation results in an increased secretion of IL-1β, a neurotoxic cytokine [255]. Since approximately half of the PLWH are above age 50 or more and with multimorbidity [50], old age, multimorbidity, SARS-CoV-2 and HIV-1 coinfection cumulatively mediate NLRP3 inflammasome overactivation, exacerbating immune dysfunction in PLWH.

### 6.2. Neurotoxic Activities of SARS-CoV-2 Proteins

The SARS-CoV-2 genome encodes four structural proteins and additional accessory proteins [256,257], including spike (S), nucleocapsid (N), envelope (E) and membrane (M) proteins and several accessory proteins (such as ORF3a) that are neurotoxic molecules [254]. These proteins primarily exert neurotoxic activities through inflammasome activation, specifically the NLRP3 inflammasome, leading to the excessive release of proinflammatory cytokines (Figure 2) [34,35,258]. A recent study using transgenic mice expressing human ACE2 showed that SARS-CoV-2 drives NLRP3 inflammasome activation in human microglia via spike protein [35]. Another study using macrophages derived from COVID-19 patients showed spike protein-induced inflammasome activation and the release of mature IL-1β [259]. In addition, SARS-CoV-2 spike protein subunit S1 was reported to exaggerate cytokine production in human PBMC [260]. Mechanistically this innate immune modulation by S1 occurs via the NFkB pathway and ROS generation, as seen in the cases of other coronaviruses [260,261,262]. S1-induced neuroinflammation was also observed in BV-2 microglial cells, where the treatment of BV-2 cells with S1 induced an increased production of IL-1β, TNF-α, IL-6 and iNOS/NO [34]. Additionally, the study showed that S1-induced neuroinflammation occurred via NFkB and p38 MAPK by increasing NLRP3 and TLR4 levels [34]. The SARS-CoV-2 spike S1 subunit was also found to induce neuroinflammatory, microglial and behavioral sickness responses via PAMPs-like mechanisms [263]. The administration of S1 protein into the mouse hippocampus induced cognitive deficit and anxiety-like behavior via IL-1β induction, illustrating its neurotoxic property [264]. 

In addition to S-protein N, E and ORF3a proteins are found to play crucial roles in modulating monocyte/macrophage activities and microglial NLRP3 inflammasome activation [242,243,258]. The SARS-CoV-2 nucleocapsid (N-protein) mechanistically binds to NLRP3 and modulates inflammasome activation [242]. The N-protein–NLRP3 interaction promotes NLRP3 binding with ASC and NLRP3 inflammasome assembly, resulting in NLRP3 activation and the induction of hyperinflammation [242]. A study using mice demonstrated that the N-protein-induced NLRP3 inflammasome activation led to the induction of IL-1β and IL-6 [242]. The NLRP3-specific inhibitor MCC950 and caspase-1 inhibitor Ac-YVAD significantly blocked the N-protein-induced NLRP3 inflammasome induction, suggesting the therapeutic potential of these inhibitors against COVID-19 [242]. N-protein was also found to be associated with the linker region of gasderminD (GSDMD) and hindered its cleavage by caspase-1, resulting in the inhibition of pyroptosis [265]. It was shown that N-protein has dual roles in regulating the host’s innate immune response. At a low dose, it suppresses IFN-1 signaling and inflammatory cytokine production. In contrast, it promotes IFN-1 signaling and inflammatory cytokine production at a higher dose [266]. These effects were achieved by regulating the phosphorylation and translocation of IRF3, STAT1 and STAT3 [266]. Interestingly, at a low dose, N-protein can bind to TRIM25 and suppress ubiquitination and the activation of RIG-I [266]. Further studies are needed to reveal how this protein modulates neuroinflammation and neurodegeneration. In comparison with the N protein, much less is known about the SARS-CoV-2 E protein. It was reported to differentially regulate the NLRP3 inflammasome response and promote the activation and release of increased IL-1β and IL-18 at the advanced stage of the disease [243].

ORF3a (viroporin) is another SARS-CoV-2 protein. It activates the NLRP3 inflammasome, leading to the expression of IL-1β via the NFkB pathway [258]. Mechanistically, this ORF3a-mediated activation of NLRP3 occurs via modulation of the potassium ion efflux [258]. ORF3a induces the oligomerization of NLRP3 and NEK7 in either an ASC-dependent or independent mode [258]. The application of NLRP3-specific inhibitor MCC950 resulted in the blockade of inflammasome activation [258], suggesting that NLRP3-specific inhibitors may be used to treat innate immune overactivation and the devastating consequences of HAND due to the SARS-CoV-2/HIV-1 syndemic [258,266]. In addition to NLRP3-specific inhibitors, IL-1β inhibitors, such as anakinra and canakinumab, may be considered an alternative to combat the severe effects of SARS-CoV-2 on HAND [267].

### 6.3. Other

One of the mechanisms behind the impact of COVID-19 on PLWH and HAND is emotional and psychological sequela, leading to neurocognitive disorder and mental health disorders [108,268]. To prevent COVID-19 from spreading, worldwide restriction measures of lockdown and social distancing were put in place [269]. With these COVID-19-imposed measures, the world population was facing loneliness, anxiety and depression [269]. As PLWH, especially PLWH with HAND, are already in compromised emotional and mental health, COVID-19-induced social restrictions may further exacerbate their mental health conditions. This psychological/emotional parameter is understudied and warrants further attention to cope with the pandemic. As COVID-19/PLWH patients can obtain information frequently from social media about the emergence of new variants with greater transmission efficiency as well as the severe outcome, this information and/or misinformation may make COVID-19/PLWH people, especially seniors, more frustrated. Therefore, social well-being, job security, economic support and other measures can help us deal with the current situation of the SARS-CoV-2/HIV-1 syndemic.

## 7. SARS-CoV-2/HIV-1 Syndemic Challenges

The SARS-CoV-2/COVID-19 pandemic has had a catastrophic impact on the world population. The COVID-19 pandemic overlaps with the preexisting HIV/AIDS epidemic, leading to COVID-19/HIV/AIDS syndemic. PLWH, especially those who have progressed to AIDS, with dysregulated/impaired immune systems, are assailable to SARS-CoV-2 infection and COVID-19 severity. The COVID-19/HIV/AIDS syndemic generated new challenges in terms of the prevention, diagnosis and treatment of these infectious diseases. Several studies showed that the current cART regimen for HIV/AIDS has some protective effects in terms of SARS-CoV-2 infection and COVID-19 severity, but it had no effects in other studies [160,270,271]. Hence, it is necessary to explore the effect of cART on PLWH to overcome the SARS-CoV-2/HIV-1 syndemic-associated neuroinflammation as SRAS-COV-2 and/or viral proteins-induced systemic inflammation may exacerbate dysregulated neuroinflammation in PLWH [122]. Nevertheless, SARS-CoV-2/COVID-19 and HIV-1/AIDS syndemic impose challenges on PLWH worldwide, including but not limited to the following:

(a). Fear among PLWH for SARS-CoV-2 infection has decreased their engagement in care. They are even scared to visit pharmacies to collect cART. This fear is heightened in the SARS-CoV-2/HIV-1 syndemic, and individuals remain unaware of how the future changes due to COVID-19 may impact the ongoing antiretroviral treatment against HIV-1.
(b). Broad-scale COVID-19-imposed lockdown measures to contain the SARS-CoV-2 infection culminated in restrictions to movements and the suspension of public and private transportation. These restrictions have hindered engagement with HIV testing services and access [272].
(c). Employment loss among migratory workers who return to their original places, primarily rural areas, is another challenge with a dual impact on PLWH. Thus, imposing a loss of contact with their primary clinic and economic losses hinders these people from availing healthcare support [273,274].
(d). The COVID-19 pandemic has overwhelmed the worldwide healthcare system. The high demand and shortage of healthcare workers enforced the engagement of HIV-1 physicians in COVID-19 care, resulting in a lack of care and routine testing for PLWH [275].
(e). The diversion of healthcare workers and facilities disrupted testing and the identification of drug resistance and opportunistic infections. This unavailability of care and enforced disruption in cART furthers the emergence of drug resistance due to the accumulation of mutations and leads to death due to opportunistic infections [36].
(f). It was speculated from multiple mathematical models that the disruption of cART may increase HIV-1/AIDS-related deaths [276].
(g). HIV-1 vaccine discovery is severely hindered due to the COVID-19 pandemic. The lockdowns, restrictions and emergency regulations owing to the pandemic clinical trials of HIV-1 vaccines are derailed, resulted in a delay in reduction ofHIV-1 vaccine discovery.
(h). The issue of vaccine hesitancy among PLWH is a primary concern. Unvaccinated people are at higher risk of SARS-CoV-2 infection and COVID-19 severity, which may worsen HAND [277]. There must be awareness and priority to vaccinate PLWH. Additionally, a lower level of anti-SARS-CoV-2 spike protein antibody was reported in PLWH compared to non-PLWH control after vaccination with mRNA-1273 or BNT126b2 [278]. The suggestion is that higher dosages and frequent booster dosages are essential for PLWH to maintain long-term immunity at the level of an average person.

## Figures and Tables

**Table 1:** Similar attributes of SARS-CoV-2 and HIV-1.

SARS-CoV-2		HIV-1
Yes	Public fear	Yes
Yes	Enveloped virus	Yes
Yes	ssRNA genome	Yes
Yes	Natural origin	Yes
No	Symptoms in the natural reservoir	No
Yes	Asymptomatic spread	Yes
Yes	Inflammation	Yes
Yes	NLRP3 inflammasome activation	Yes
Yes	Lymphopenia	Yes
Yes	NETosis	Yes
Yes	Neurocognitive disorders	Yes

**Table 2:** Differences between SARS-CoV-2 and HIV-1 (adapted from Ref. [42] with modifications).

	SARS-CoV-2	HIV-1
Phylogeny	β-coronavirus	Lentivirus
Virion size	Spherical particle 50–200 nm in diameter	Spherical particle ~100 nm in diameter
Genome	One copy of single-stranded positive-sense RNA	Two copies of single-stranded positive-sense RNA
Genome size	~29.2 kb	~10 kb
Genome integration	No	Yes
Reservoir	No viral reservoir formation	This virus integrates into the human genome and forms reservoirs
Transmission	Air, aerosol	Sexual, body fluid
Receptor in use	ACE2, TMPRSS2	CD4, CCR5 and CXCR4
Symptoms	Breathing difficulty, fever, pneumonia and kidney failure	Flu or mononucleosis-like during early infection and opportunistic infections in late stages. Long time to progress to AIDS
Symptom timeline	2–14 days after contact with the virus	2–6 weeks after viral contact
Death percentage	1–4%	≥95%
Vaccine	Available	Not available
Medicine	Antivirals	Antiretroviral therapy (cART)
Cure	Curable (By antivirals or plasma therapy)	No cure (Can be controlled with cART)

## Data Availability

Data are available from corresponding authors upon reasonable request.
